# Formulation of Ready-to-Use Broccoli Extracts Rich in Polyphenols and Glucosinolates Using Natural Deep Eutectic Solvents

**DOI:** 10.3390/molecules29235794

**Published:** 2024-12-07

**Authors:** Ivona Karaula, Emma Vasung, Anja Damjanović, Manuela Panić, Mia Radović, Kristina Radošević, Martina Bagović Kolić, Marina Cvjetko Bubalo, Ivana Radojčić Redovniković

**Affiliations:** Faculty of Food Technology and Biotechnology, University of Zagreb, 10000 Zagreb, Croatiaadamjanovic@pbf.hr (A.D.); mradovic@pbf.hr (M.R.); krado@pbf.hr (K.R.); mbagovic@pbf.hr (M.B.K.); iradojci@pbf.hr (I.R.R.)

**Keywords:** natural deep eutectic solvents, green extraction, broccoli, polyphenols, glucosinolates, HaCaT cells

## Abstract

Broccoli is rich in biologically active compounds, especially polyphenols and glucosinolates, known for their health benefits. Traditional extraction methods have limitations, leading to a shift towards using natural deep eutectic solvents (NADESs) to create high-quality extracts with enhanced biological activity. This study focuses on preparing broccoli extracts in NADES, enriched with polyphenols and glucosinolates, without additional purification steps. Using the COSMOtherm software, the solubility of polyphenols and glucosinolates in NADESs was predicted, and five biocompatible betaine-based NADESs were prepared with glucose (B:Glc_1:1_ and B:Glc_5:2_), sucrose (B:Suc), glycerol (B:Gly), and malic acid (B:MA) as hydrogen bond donors. The resulting extracts were assessed for total polyphenol and glucosinolate content, along with antioxidant capacity, using the ORAC assay. The results demonstrated that NADES extracts contained higher polyphenol content and exhibited enhanced antioxidant effects compared to the reference ethanol extract, with B:Glc_1:1_ extract showing the highest performance among all the extracts tested. On the other hand, the extract based on B:MA exhibited nearly six times higher total glucosinolate content compared to the ethanol extract. Additionally, polyphenols and glucosinolates were generally more stable in NADES extracts than in the reference solvent. Finally, the B:Glc_1:1_ extract, identified as optimal in terms of polyphenol and glucosinolate content and stability, exhibited mild stimulation of HaCaT cells growth and facilitated the wound-healing process. Through green chemistry parameter calculations, we demonstrated that the extraction of broccoli bioactives using B:Glc_1:1_ can be considered sustainable, underscoring the potential of NADESs for producing ready-to-use plant extracts.

## 1. Introduction

Among plant-based foods that are rich in biologically active compounds, species of the genus *Brassica*, particularly broccoli, are of significant importance. In addition to being a source of fiber and essential minerals, broccoli is a natural reservoir of glucosinolates, phenols, vitamins, and carotenoids [1]. Glucosinolates, a complex group of thioglucoside compounds, are particularly noteworthy due to their degradation products, such as isothiocyanates, thiocyanates, nitriles, epithionitriles, and oxazolidine-2-thione, that are formed under biotic and abiotic stress in reactions catalyzed by myrosinase and have demonstrated antitumor properties. Notably, sulforaphane, derived from glucoraphanin, and indole-3-carbinol, a hydrolysis product of indolic glucosinolate glucobrassicin, have been linked to cancer prevention [2,3]. Broccoli also contains a diverse range of phenolic compounds, with over 8000 naturally occurring variants characterized by the presence of one or more hydroxylated benzene rings. These phenolic compounds are crucial due to their potent antioxidant activity and their overall positive effects on human health. Flavonoids, a major subgroup of phenolic compounds, exhibit the strongest antioxidant properties [4]. Additionally, polyphenols in broccoli have been shown to possess antitumor and vasodilatory properties, further contributing to its classification as a functional food [5]. Broccoli extract has also gained recognition as a valuable ingredient in skincare formulations due to its antioxidant and anti-inflammatory properties, which strengthen cellular defenses against oxidative stress and support skin health. Moreover, topical application of broccoli sprout extract has shown protective effects against UV-induced damage, with studies in humans and mice demonstrating significantly reduced UV-induced erythema, a key marker of skin inflammation and damage, in treated areas [6].

Due to the various drawbacks associated with conventional methods for extracting bioactive compounds from plant materials, such as low efficiency, degradation of sensitive compounds, and the use of toxic solvents, recent trends have shifted towards obtaining high-quality extracts that exhibit enhanced biological activity and extended shelf life while minimizing the use of harmful solvents [7]. In this context, deep eutectic solvents (DESs) have emerged as promising green alternatives. DESs, eutectic mixtures of hydrogen bond donors (HBDs) and acceptors (HBAs), exhibiting enthalpy-driven negative deviations from thermodynamic ideality [8], offer advantages such as low toxicity, biodegradability, and a high degree of tunability and ability to solubilize versatile organic compounds, making them suitable solvents for the extraction of bioactive compounds [9]. Over the past 15 years, DESs have been effectively employed to extract bioactive compounds, particularly polyphenols, from a diverse range of sources, including fruits, vegetables, medicinal herbs, and food processing residues [10]. However, challenges remain, particularly regarding the complete removal of DES residues from final products. Namely, the non-volatile nature of DESs complicates this removal process, posing significant challenges to sustainability and regulatory compliance [9]. However, natural DESs (NADESs), a subclass of DESs derived exclusively from natural components (e.g., methylamines choline and betaine, sugars, organic acids, and amino acids), are speculated to be safe for human consumption, meaning that NADESs can be utilized to prepare liquid extracts, referred to as ready-to-use extracts, which can be directly applied in food, pharmaceuticals, and cosmetics [11]. Furthermore, due to their high bioactive solubilization ability, antimicrobial and antioxidative properties [12,13], and capacity to stabilize bioactives, NADESs can serve not only as extraction media, but also as excellent storage media [9,14].

Another key advantage of using DESs in the extraction of biologically active compounds is their tunability: by varying the components that form the DES and adjusting the water content, these solvents can be tailored to meet the specific requirements of a given extraction process. The vast chemical diversity of DESs offers extraordinary potential for solvent design but also presents a significant challenge in identifying the “ideal” DES that fulfils various predefined criteria. Selecting suitable DESs from the nearly limitless combinations available can be a time-consuming and costly process when relying solely on traditional trial-and-error methods. To overcome these challenges, recent efforts have focused on the rational design of DESs using computational techniques, which significantly expedite the screening process [15]. Among these methods, Conductor-like Screening Model for Real Solvents (COSMO-RS) has emerged as one of the most accurate ab initio computational approaches for ranking solvents. COSMOtherm, software that implements this approach to predict solvent properties and interactions, serves as an efficient pre-screening tool, reducing the need for extensive experimental testing and accelerating the identification of optimal DESs [16]. Recently, this software has been successfully utilized to predict the solubility of phenolic diterpenes from the aromatic herb rosemary (*Rosmarinus officinalis* L.) [17], and phenolics from the medicinal herb *Teucrium chamaedrys* [18], grape pomace from the Graševina variety [15], and leaves of Ginkgo (*Ginkgo biloba* L.) [19] in DESs. This predictive capability has facilitated the preparation of extracts rich in these components, that have high antioxidant activity.

The application of (NA)DESs for the extraction of polyphenols from broccoli has been successfully demonstrated in several instances [20,21,22,23]. In contrast, the potential of (NA)DESs for extracting glucosinolates from plants, including broccoli, remains entirely unexplored. Therefore, given the health benefits of polyphenols and glucosinolates found in broccoli, along with the numerous advantages of NADES-based ready-to-use plant extracts, this study aimed to develop high-value, stable liquid extracts of broccoli in NADESs, enriched with these two groups of compounds, for direct application in the cosmetic industries.

## 2. Results and Discussion

To develop NADES-based broccoli extracts rich in polyphenols and glucosinolates, we selected optimal solvents through computer-aided screening, analyzed the bioactive content and stability of the obtained extracts, and assessed the bioactivity of the most promising extract for potential skin-related applications. Additionally, the extraction process was evaluated for sustainability using green chemistry parameters.

### 2.1. In Silico Pre-Selection of NADES

**Figure 1:**
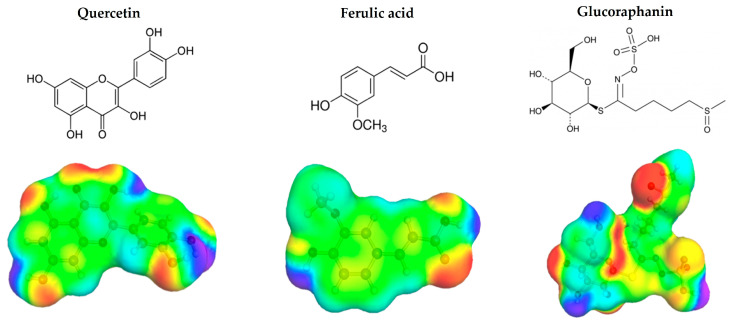
Structures of quercetin, ferulic acid, and glucoraphanin used as input parameters for COSMOtherm calculations.

The solubility of polyphenols and glucosinolates was evaluated in 50 different NADESs using the COSMOtherm software. These NADES included conventional formulations based on cholinium chloride and betaine as HBAs, paired with various HBDs such as polyols, sugars, and organic acids, as well as combinations of organic acids or amino acids with sugars. Additionally, varying water contents, up to 50% (*w*/*w*), were considered. Higher dilutions of NADESs were not included, as additional water would result in a solvent behaving more like a solution of their components in water [24]. Polyphenols and glucosinolates encompass a diverse range of compounds; therefore, for simplicity in simulations, quercetin, a widely studied flavonoid aglycone, and ferulic acid, a commonly studied phenolic acid, both present in broccoli in notable amounts [25], were selected as representative polyphenols. Moreover, quercetin is widely studied in the context of human health and nutrition, which makes it a valuable marker for the assessment of phenolic extraction efficiency [26]. Additionally, glucoraphanin, the most significant glucosinolate in broccoli [2,3], was included for solubility evaluation. Figure 1 illustrates the structures of quercetin, ferulic acid, and glucoraphanin and their corresponding *σ*-surfaces after geometrical optimization. The green, red, and blue regions correspond to the non-polar “neutral” part of the molecules; the “hydrogen acceptor” region, with a negative charge; and the “hydrogen donor” region, with a positive charge, respectively [27]. The data for each molecule is contained in a corresponding .cosmo file used as the input parameters for the computational solubility simulations.

The output parameter of the COSMOtherm solubility assessment is the logarithm of the activity coefficient at infinite dilution, ln(*γ*), which indicates the solubility of the examined compound in the chosen solvent. A lower activity coefficient signifies greater solubility of the investigated molecule in the observed solvent. The ln(*γ*) of quercetin, ferulic acid, and glucoraphanin are graphically presented in Table 1. The accompanying scale in Table 1 indicates that in the search for a solvent that can best dissolve the desired compound, one should aim for values in the interval [−∞; 0] [15]. According to the data presented in Table 1, all the chosen NADESs are, in general, suitable solvents for dissolving the selected polyphenols and glucosinolates. For all the selected polyphenols and glucosinolates, a clear trend of decreasing solubility was observed with increasing water content. This aligns with findings in the literature [15], leading to the conclusion that precise control over the water content in (NA)DESs significantly influences the efficacy of the extraction process.

Based on the calculated ln(*γ*) values, betaine-based NADESs generally exhibited superior solubilization of the target bioactives compared to both choline chloride-based alternatives and NADESs composed of methylamine-free components, such as combinations of organic acids or amino acids with sugars. Therefore, NADESs consisting of betaine combined with glucose, sucrose, glycerol, or malic acid, each recognized as safe for human consumption, were selected for further research. Moreover, betaine exhibits therapeutic and biological effects that may be beneficial in alleviating a wide range of human diseases and conditions [28]. As mentioned, in silico screening of NADESs showed that those with 10% water (*w*/*w*) exhibit superior solubility for the target compounds. However, such NADESs tend to be highly viscous [29] and, in our experience, can even crystallize over time, making them difficult to handle and problematic for extraction and filtration processes. Therefore, formulations with 30% water were considered optimal for ensuring both effective extraction and manageable operational conditions in subsequent research.

### 2.2. Preparation and Characterization of NADES

Based on the in silico pre-selection, five betaine-based NADESs, with glucose, sucrose, glycerol, or malic acid as the hydrogen bond donor, were prepared and characterized (Table 2).

Sugar- and acid-based NADESs exhibited similarly high viscosities, ranging from 54.13 to 71.49 mPa·s, with densities exceeding 1.19 g cm^−3^. In contrast, the polyol-based NADES had the lowest values for both properties, with a viscosity of 25.70 mPa·s and a density of 1.17 g cm^−3^. The measured pH values of most of the NADESs fell within the neutral range (7.03–7.85). The only significant deviation was observed for B:MA, which had a pH of 3.24. This is expected, as the pH of a (NA)DES is primarily related to the nature of its components, and a (NA)DES containing an organic acid typically results in an acidic pH. Furthermore, the molar transition energy (ENR), a measure of polarity, ranged from 49.21 to 49.90 kcal mol^−1^ across the NADESs, with lower values indicating higher polarity. All NADESs tested were more polar than the reference solvent (51.06 kcal mol^−1^. The highest polarity was recorded for B:MA (49.21 kcal mol^−1^). These findings align with research by Dai et al. [30], which reported that organic acid-based NADESs are generally the most polar, while those containing sugars and polyols exhibit lower polarity. It is crucial to mention that for conventional extraction solvents it is well established that some polyphenolic compounds degrade under high-pH conditions [31], while glucosinolates are prone to degradation at pH values below 4 [32]. On the other hand, the optimal extraction of polyphenols generally requires acidic to slightly acidic conditions [33,34]. Balancing these conditions is therefore essential to effectively extract and stabilize both polyphenols and glucosinolates. However, unlike conventional solvents, (NA)DESs do not behave in the same way regarding pH. The pH of NADESs is not always the most critical factor for application outcomes. Instead, other solvent properties, such as polarity, viscosity, and specific interactions with bioactive compounds, often play a more decisive role. More precisely, we have previously shown that for stabilizing enzymes [35] or coenzymes [36] the pH of DESs does not necessarily align with aqueous pH optima for biomolecule stability. This unique behavior suggests that the properties of NADESs can compensate for pH limitations in certain contexts. This rationale underpins our investigation into the extraction efficiency and stabilization capacity of NADESs across a range of pH values, without being constrained by the existing literature on the behavior of target bioactives in conventional solvents.

Finally, for the preliminary evaluation of cytotoxicity and proof of biocompatibility, NADESs demonstrated no growth inhibition on the human keratinocyte cell line, HaCat. The EC_50_ values were determined to be greater than 2000 mg L^−1^ (Table 2), indicating that NADESs have good biocompatibility with human skin.

### 2.3. Characterization of Extracts

NADES-based broccoli extracts were evaluated for polyphenol and glucosinolate content and stability, as well as for antioxidant capacity. A 70% ethanol (*v*/*v*) solution was used as the reference solvent due to its common use in the extraction of bioactive compounds, allowing for comparison with conventional extraction methods. It is important to note that using methanol as an extraction solvent could result in a higher yield of glucosinolates [37]. However, its toxicity makes it unsuitable for use in extracts intended for human application, and it was therefore not considered as a reference solvent.

#### 2.3.1. Content of Bioactives

**Figure 2:**
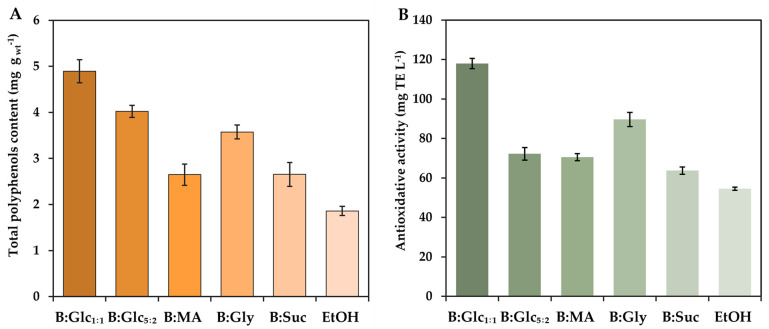
(**A**) Total polyphenolic content in the prepared broccoli extracts. (**B**) ORAC values of the prepared extracts. Results are expressed as the means (*n* = 3) ± S.D.

Based on the results shown in Figure 2A, all NADES formulations demonstrated superior polyphenol extraction efficiency compared to ethanol, with yields ranging from 2.65 to 4.90 mg g_dw_^−1^. The glucose-based NADESs, particularly B:Glc_1:1_, achieved the highest polyphenol content (4.90 mg g_dw_^−1^), representing a more than 2.5-fold increase over the reference solvent (1.86 mg g_dw_^−1^). These findings highlight the predictive accuracy of the COSMOtherm software in selecting solvents for polyphenol solubilization and are in line with the work of Ianni et al. [38], which demonstrated the superior efficiency of NADESs over conventional organic solvents for polyphenols extraction. Furthermore, studies have indicated that certain NADESs, including betaine-based solvents, outperform traditional solvents in extracting flavonoid glycosides, aglycones, and phenolic acids [20,39]. Yoo et al. [40] also observed that NADESs provided up to a twofold increase in extraction efficiency for polyphenols compared to conventional methods using water or aqueous organic solvents. This can be explained by the unique properties of (NA)DESs. These solvents, including betaine-based ones, have a remarkable ability to solvate polyphenols due to interactions such as hydrogen bonds, dipole–dipole interactions, and van der Waals forces. These strong hydrogen bond interactions effectively overcome polyphenol–polyphenol interactions, which enhances the dissolution and diffusion of polyphenols out of plant cells [41]. Additionally, it has been demonstrated that DESs can significantly disrupt the plant cell wall structure, which facilitates faster extraction of the target compounds. For instance, SEM and FE-SEM imaging of orange peel extractions has shown that these solvents can break down cell walls more efficiently, leading to quicker and more effective extraction of phenolic compounds [42,43,44].

**Figure 3:**
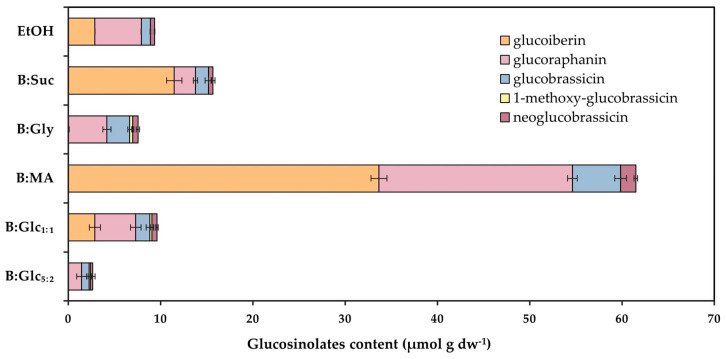
Glucosinolate profile of NADESs and ethanol (70%, *v*/*v*) extracts of broccoli. Results are presented as mean ± S.D. (*n* = 3).

The glucosinolates identified in the extracts included the aliphatic glucosinolates glucoiberin and glucoraphanin, as well as the indole glucosinolates glucobrassicin, 1-methoxy-glucobrassicin, and neoglucobrassicin (Figure 3). The total glucosinolate content varied significantly across NADES extracts, ranging from 7.55 µmol g_dw_^−1^ in B:Gly to 61.50 µmol g_dw_^−1^ in the B:MA extract. Notably, the B:MA extract showed nearly six times higher total glucosinolate content compared to the ethanol extract (9.36 µmol g_dw_^−1^). It also contained the highest levels of each individual glucosinolate, except for 1-methoxy-glucobrassicin, which was not detected in this extract. Additionally, aliphatic glucosinolates were consistently more abundant than indole glucosinolates in all extracts, in line with the literature that identifies the aliphatic glucosinolates glucoraphanin and glucoiberin as the predominant glucosinolates in broccoli [45]. Among the indole glucosinolates, glucobrassicin was the most prevalent, which also agrees with reports showing it accounts for about 60% of indole glucosinolates and roughly 10% of total glucosinolate content [46]. The B:MA extract exhibited a notably high concentration of glucoraphanin (20.95 µmol g_dw_^−1^), the predominant glucosinolate in broccoli [46] and a key precursor of sulforaphane, a compound extensively studied for its chemoprotective properties [2]. This concentration reflects nearly a fourfold increase compared to the alcohol extract, which contained only 5.04 µmol g_dw_^−1^ of glucoraphanin.

#### 2.3.2. Stability of Bioactives

**Figure 4:**
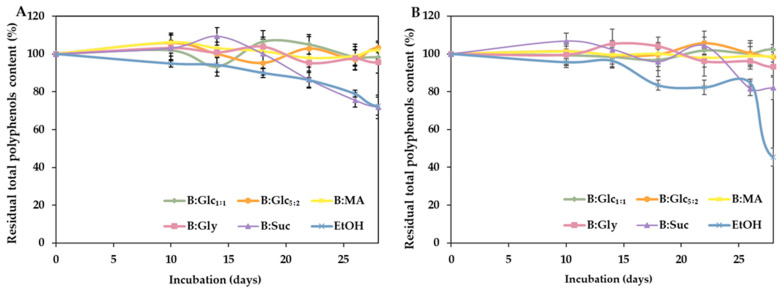
Residual concentration of total polyphenols in broccoli extracts: polyphenol content in extracts stored at 4 °C (**A**) and 25 °C (**B**), expressed as the ratio of polyphenols concentration in the extract after incubation and the initial polyphenol concentration in the extract. Results are presented as mean ± S.D. (*n* = 3).

**Figure 5:**
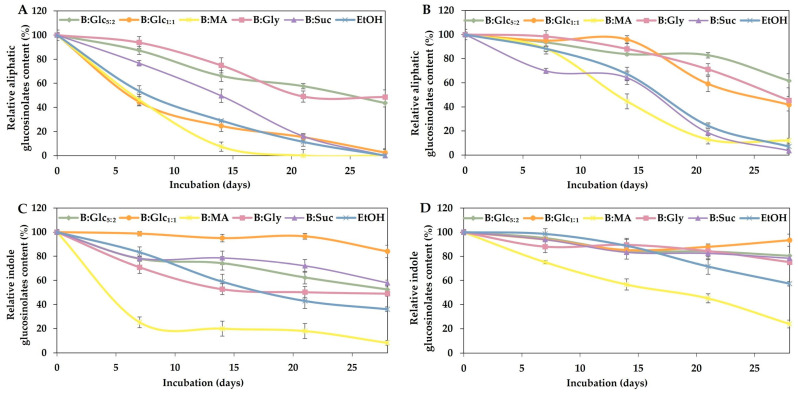
Residual concentration of glucosinolates in broccoli extracts: aliphatic glucosinolates in extracts stored at 25 °C (**A**) and 4 °C (**B**), and indole glucosinolates in extracts stored at 25 °C (**C**) and 4 °C (**D**), expressed as the ratio of glucosinolate concentration in the extract after incubation to the initial glucosinolate concentration in the extract. Results are presented as mean ± S.D. (*n* = 3).

The stability of polyphenols and glucosinolates in the extracts was monitored over 28 days at both room temperature (25 °C) and refrigerated conditions (4 °C), and the results are shown in Figure 4 and Figure 5.

Stability measurements at both temperatures revealed similar trends in the residual concentration of total polyphenols across the selected NADESs: B:Glc_5:2_, B:Glc_1:1_, B:Gly, and B:MA were highly effective in preserving polyphenols, with residual concentrations exceeding 90% after a 28-day incubation. In comparison, residual polyphenol concentrations in the ethanol-based extract dropped to 72% at 4 °C and 45% at room temperature after a 28-day incubation period (Figure 4). These findings are consistent with previous research by [30], which also reported superior stability of cyanidin polyphenols in NADESs compared to acidified ethanol. Stability of polyphenols during long-term incubation up to 80 days in NADESs was also reported by Mitar et al. [47]. The stabilization ability of NADESs is often attributed to the interactions between the solvent and the target biomolecules, primarily through hydrogen bonding. These interactions slow down the movement of biomolecules and reduce their exposure to oxygen, thereby minimizing oxidative degradation [30,48,49]. In addition, our recent findings have demonstrated a significant positive correlation between the density/viscosity of DES-based systems and their effectiveness in stabilizing biomolecules, such as nicotinamide coenzymes [50]. This stabilization effect can be linked to the slower molecular dynamics of these solvents, which create a less disruptive environment for sensitive compounds. In this context, while the high viscosity and density of (NA)DESs are often seen as drawbacks, these properties may actually be advantageous for maintaining the stability of a bioactive in a liquid extract by limiting degradation and preserving their activity over time.

As shown in Figure 5, the stability of glucosinolates varied among the extracts, with results indicating that indole glucosinolates generally exhibited greater stability than aliphatic glucosinolates. As for aliphatic glucosinolates, at 4 °C the B:Glc_5:2_ extract exhibited superior stability, retaining 62% of the initial glucosinolate concentration after 28 days, while the reference solvent maintained only 7% of the initial concentration over the same period. In contrast, substantial degradation of aliphatic glucosinolates was observed in the B:Suc and B:MA extracts, which was comparable to the degradation seen in the reference extract. At 25 °C, the B:Glc_5:2_ and B:Gly extracts demonstrated the highest stability for aliphatic glucosinolates, retaining 44% and 48% of their initial concentrations, respectively. In contrast, the other extracts exhibited complete degradation of aliphatic glucosinolates after 28 days, similar to the reference extract. For indole glucosinolates, the B:Glc_1:1_ extract displayed the highest retention, with concentrations reaching 93% at 4 °C and 84% at 25 °C after 28 days. In contrast, the concentration of indole glucosinolates in the reference solvent decreased to 57% at 4 °C and 36.1% at 25 °C during the same period. Furthermore, B:Glc_5:2_, B:Suc, and B:Gly retained over 49% of their initial indole glucosinolate concentrations regardless of the incubation temperature. In contrast, similar to the observations for aliphatic glucosinolates, indole glucosinolates experienced substantial degradation in the B:MA extract, with residual concentrations falling below 24.01% at both incubation temperatures. This finding aligns with the work of Jing et al. [32], who reported that glucosinolates are relatively unstable at pH < 4 (pH value of B:MA was 3.25; Table 2).

#### 2.3.3. Antioxidative Activity

The antioxidant capacity of the broccoli extracts was measured using the ORAC assay, with the results shown in Figure 2B. A key advantage of this assay is that it operates under physiological conditions, mimicking those found in the human body. Additionally, it generates radical species that simulate lipid peroxyl radicals involved in the peroxidation of biological components [51]. All NADES-based extracts demonstrated ORAC values greater than that of the ethanol extract, with the B:Glc_1:1_ extract exhibiting the highest antioxidant capacity, yielding an ORAC value of 117.97 mg TE L_ext_^−1^—approximately a 2-fold increase compared to the reference ethanol extract, which had an ORAC value of 54.53 mg TE L_ext_^−1^. As anticipated based on prior studies by [15] and [52], these antioxidant capacity results are closely correlated with the polyphenol content (Figure 2A).

#### 2.3.4. Trades-Off Between the Extracts with Respect to Target Properties

As mentioned, the goal of this study was to develop a stable, ready-to-use broccoli extract rich in two key classes of bioactive compounds—polyphenols and glucosinolates. To achieve this, we established performance targets based on the bioactive content, stability, and biological activity of the extracts, aiming to identify a balanced formulation that met these criteria.

**Figure 6:**
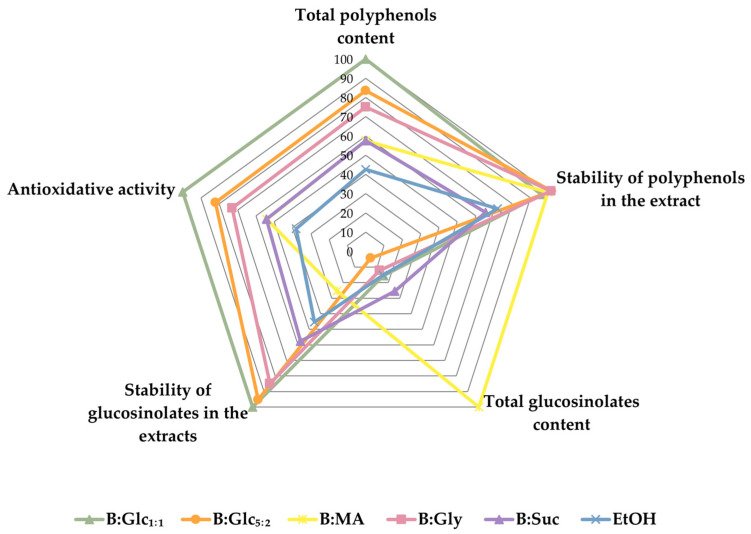
Radar plot evaluating the extracts in terms of target properties. The radar chart is bounded by the specific lower and upper limits for each target property. Ratings, ranging from 0 to 100, reflect the performance of extracts relative to the best candidate for each target property.

Our evaluation revealed that different performance targets led to conflicting conclusions regarding the optimal extract. For instance, the B:MA extract exhibited the highest total glucosinolate content (Figure 4), but its polyphenol content and antioxidant capacity were only average, comparable to the reference ethanol extract (Figure 2). Additionally, in the B:MA extract glucosinolates were the most unstable during storage, showing significant degradation during the incubation period (Figure 5). On the other hand, the B:Glc_1:1_ extract demonstrated the highest total polyphenol content and ORAC value (Figure 2), but contained glucosinolates at levels similar to the ethanol-based extract (Figure 4). However, the B:Glc_1:1_ extract offered the best glucosinolate preservation (in terms of total glucosinolates), regardless of the storage conditions. These findings indicate that achieving an optimal extract requires balancing bioactive content, stability, and biological activity, as illustrated in Figure 6, which highlights the trade-offs between these performance properties.

As can be seen from Figure 6, B:Glc_1:1_ showed the most balanced distribution across all target property values. This particular NADES outperformed the others in terms of total polyphenol content, ORAC value, and glucosinolate stability. While it matched the top candidates in polyphenol stabilization, its performance in extracting glucosinolates was only average. Given our objective to produce stable liquid broccoli extracts, the exceptional stabilization properties of this NADES were considered the most critical factor, leading us to select it as the best candidate.

#### 2.3.5. Evaluation of Keratinocyte Response to B:Glc_1:1_-Based Broccoli Extract

Given the biocompatibility of the NADES used (Table 2) and the skin-beneficial properties of plant extracts, we further explored the potential of the B:Glc_1:1_ broccoli extract for topical application. The extract’s biological activity was assessed in vitro using human keratinocyte cells (HaCaT) through a proliferation assay and a scratch assay to evaluate its wound-healing effects.

**Figure 7:**
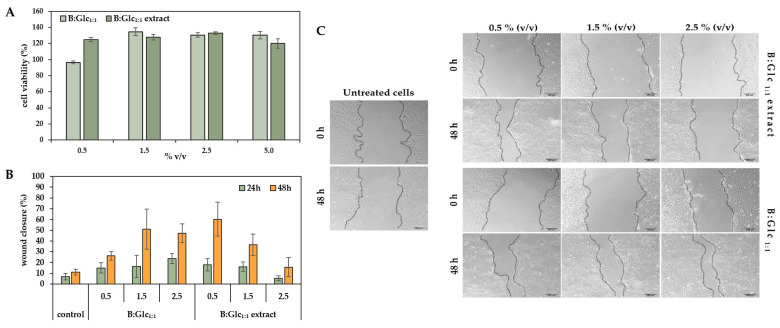
(**A**) Effect of B:Glc_1:1_ and corresponding extract on HaCat cell viability determined by the MTS assay in volume ratio 0.5–5% (*v*/*v*). (**B**) Migration assessment of HaCaT cells: the percentage of wound closure, determined from changes in gap width from the initial scratch over 24 and 48 h. (**C**) Microscopic images from in vitro scratch wound-healing assays showing cell migration into the cell-free gap (outlined) over time, comparing untreated cells as control with cells treated with B:Glc_1:1_ broccoli extract and B:Glc_1:1_ alone.

Cell survival remained high across all tested concentrations of the extract in B:Glc_1:1_ (0.5–5% *v*/*v*), as well as in B:Glc_1:1_ alone, demonstrating that neither the extract nor the NADES components were cytotoxic to HaCaT cells, with the results presented in Figure 7A. In fact, the extract showed a slight stimulatory effect on cell proliferation, suggesting it may be safe for human use. These findings align with previous research of Panić et al. [15], which found that an NADES-based grape pomace extract similarly stimulated HaCaT cell growth, with the effects depending on the concentration of polyphenols in the extract.

The same cell line was also used to test the wound-healing effects of the B:Glc_1:1_-based broccoli extract through a scratch assay to assess factors influencing cell migration and wound healing. In this assay, a controlled gap was created in a HaCaT keratinocyte monolayer, mimicking a wound, to examine cell migration—a critical phase of wound healing. Keratinocytes, as the primary skin cells, are essential for effective wound closure due to their capacity for proliferation and migration [53]. In this study, HaCaT cells treated with B:Glc_1:1_ extracts at varying concentrations (0.5, 1.5, and 2.5% *v*/*v*) were compared to cells treated with B:Glc_1:1_ alone and to untreated cells, which served as a control to evaluate the progress of wound closure, measured by the decrease in the gap width over time (Figure 7B). After 24 h, cells treated with the extract at concentrations of 0.5% and 1.5% (*v*/*v*) demonstrated a moderate increase in wound closure, reaching approximately 15–20%, compared to 5–10% in untreated cells, suggesting an enhancement in cell migration and wound healing. By 48 h, cells treated with 0.5% (*v*/*v*) extract showed the most pronounced wound closure, achieving 45–70% closure, whereas untreated cells closed only 10–15% of the wound area. Interestingly, treatment with a higher concentration of 2.5% (*v*/*v*) extract produced an inhibitory effect on cell migration, with only 15% wound closure after 48 h, indicating a concentration-dependent impact on healing. In contrast, cells treated with B:Glc_1:1_ alone exhibited optimal migration at a concentration of 1.5% (*v*/*v*) without the inhibitory effect observed at higher concentrations when compared to corresponding extract. The results are not surprising considering components of said NADES: glucose serves as a primary energy source for cell growth and proliferation, while betaine is known for its wound-healing effects and is commonly used in topical applications [54]. These findings confirm that NADESs function not merely as passive solvents but as active storage media with intrinsic bioactive properties [9], distinguishing them significantly from conventional extraction solvents. The bioactive compounds in broccoli also enhanced cell migration, but this effect was dose-dependent. At a lower extract concentration (0.5% *v*/*v*), these bioactives significantly promoted wound closure, whereas higher concentrations impaired cell migration. This suggests that while broccoli bioactives aid wound healing, excessive concentration may hinder the process, with 0.5% (*v*/*v*) proving to be an effective dose. It should be noted that while the wound closure effect is clearly visible after 48 h (Figure 7C), it is primarily concentrated at the ends of the gap, which mimic the edges of a wound. In the central region of the gap, cell proliferation and migration are less pronounced, resulting in slower closure compared to the edges.

### 2.4. Sustainability Assessment of the B:Glc_1:1_ Broccoli Extract Preparation Method

The sustainability of processes using NADESs, like the extraction of bioactive compounds investigated here, hinges on the solvent’s green properties: negligible volatility, non-flammability, non-toxicity, and easy preparation from renewable materials [9]. However, to promote their use in industrial applications, a holistic sustainability assessment of processes implementing these solvents is essential. Here, we evaluated the sustainability of a B:Glc_1:1_-based ready-to-use broccoli extract preparation method by calculating green chemistry metrics and compared the metrics to a conventional procedure using ethanol (70%, *v*/*v*) as an extraction solvent.

The sustainability factors for the extraction process were calculated based on the extraction of bioactive compounds (total polyphenols and glucosinolates) from 1 g of broccoli using 10 mL of solvent [55]. It should be noted that in comparing conventional extraction with NADES-assisted extraction, the primary extraction step was performed similarly in both methods: heating at 75 °C for 10 min for myrosinase inactivation, followed by ultrasound treatment for 50 min. However, the procedures diverge significantly in terms of downstream processing complexity. In the conventional extraction process, the solvent (70% ethanol, *v*/*v*) has to be removed under reduced pressure after extraction, yielding a solid, with the possibility of regenerating ethanol at an efficiency of *η* = 60% [9]. In contrast, NADES-assisted extraction eliminates the need for downstream processing, as the components of the NADESs used in this study, like those in other NADES formulations, are part of our daily diet and found in dietary supplements already available on the market.

According to the results shown in Table 3, it can be concluded that the process of obtaining a liquid broccoli extract using NADESs results in significantly lower sustainability factors than the conventional process, as no waste is generated. Specifically, the E-factor and EQ values for this process are 0 kg kg^−1^, while for the conventional process both values are 800 kg kg^−1^ (the environmental hazard quotient for ethanol is 1). Regarding the PMI coefficient, the extraction using NADESs (1100 kg kg^−1^) also shows better sustainability than the conventional process (2000 kg kg^−1^) since a greater quantity of plant material, and consequently extraction solvent, is needed to obtain the same amount of biologically active compounds. Considering the economic efficiency of the compared processes, the cost of NADESs required to obtain 1 kg of biologically active compounds is lower than the cost of ethanol for obtaining the same quantity of biologically active compounds, making extraction using NADESs economically viable. Although the price of NADESs (EUR 125 kg^−1^) is higher compared to the price of ethanol (EUR 80 kg^−1^) (both cases consider the commercial prices of chemicals with a purity of >99.5%), a greater amount of ethanol is required compared to NADESs to obtain the same quantity of biologically active compounds.

Although the calculated green chemistry parameters and the cost of the solvent required to obtain 1 kg of the compounds of interest indicate that the extraction process using NADESs is more environmentally and economically favorable, it should be mentioned that, when it comes to scaling up the extraction process with NADESs, the viscosity of these solvents can pose a problem and lead to additional costs associated with the energy consumption needed to ensure the homogeneity of the mixture during extraction and during the filtration of the extract [9]. Specifically, the viscosity of the NADES used herein is 71.49 mPa·s (Table 2), which is significantly higher compared to the viscosity of ethanol (1.20 mPa·s). Finally, it is important to note that one of the main advantages of NADESs is the safety during handling: the characteristics of NADESs, such as non-flammability, non-toxicity, and low volatility, make them a safer solvent compared to relatively toxic and easily flammable ethanol.

Overall, to highlight the advantages and challenges of NADES-based ready-to-use extracts for application in industry, as observed in this study and supported by the available literature, an SWOT analysis (strengths, weaknesses, opportunities, threats) was conducted (Table 4).

The primary strengths of utilizing NADESs include their remarkable flexibility and biocompatibility. These solvents can be tailored to meet specific requirements, enabling the extraction of a diverse array of bioactive compounds. Furthermore, NADESs serve as “active” solvents, contributing beneficial properties, such as antioxidant and antimicrobial effects, to the final product, thereby enhancing its overall efficacy and value. Despite these strengths, there are notable weaknesses that need to be addressed. One significant challenge lies in scaling up the production of NADESs and NADES-based products from laboratory settings to industrial applications. This transition demands precise control over formulation to ensure consistency and quality in the final product. The growing demand for natural and sustainable products creates a favorable market for NADES-based extracts, with potential for innovative applications through collaboration between academia and industry. Advancements in regulatory frameworks could help promote the use of NADES-based systems, opening up more opportunities for industrial applications. However, growing competition in research and the development of alternative extraction methods present challenges. Navigating strict safety and quality regulations is also important. While NADESs show promise for extracting bioactive compounds, tackling these challenges and taking advantage of new opportunities is key for their successful use in the life sciences industry.

## 3. Materials and Methods

### 3.1. Chemicals and Materials

NADES components, including DL-malic acid (≥98%) and sucrose (≥99.5%), were purchased from Sigma-Aldrich (St. Louis, MO, USA), while glycerol (anhydrous) and Folin–Ciocalteu’s phenol reagent were obtained from Merck (Darmstadt, Germany). D-glucose anhydrous, (≥99.5%) and betaine (anhydrous, 98%) were sourced from Acros Organics (Geel, Belgium), and 96% ethanol was acquired from Kemika (Zagreb, Croatia). All of the above-mentioned chemicals were used without further purification.

The normal human keratinocyte cell line, HaCaT, was purchased from CLS Cell Lines Service GmbH (Eppelheim, Germany). Dulbecco’s modified Eagle’s medium (DMEM) was purchased from Capricorn Scientific GmbH (Ebsdorfergrund, Germany), fetal bovine serum (FBS) was purchased from GIBCO by Life Technologies (Paisley, UK), and trypsin-EDTA was purchased from Sigma-Aldrich (St. Louis, MI, USA). Cells were cultured in BioLite petri dishes (Thermo Fisher Scientific, Drive Rochester, NY, USA) in a humidified atmosphere with 5% CO_2_ at 37 °C in the incubator. Individual experiments were performed in 96-well plates (Thermo Fisher Scientific, USA). The CellTiter 96^®^ AQ_ueous_ One Solution Cell Proliferation assay was purchased from Promega (Madison, WI, USA), while measurements were performed on the microplate reader (SpectraMax ABS Plus, San Jose, CA, USA).

Freshly harvested broccoli florets were procured from local markets in Zagreb, Croatia, and promptly transported to the laboratory for freeze-drying. The florets were finely chopped, lyophilized, and subsequently ground into a fine powder. The resulting powder was stored in a desiccator under controlled conditions until further analysis.

The software BIOVIA COSMOtherm 2020 version 20.0.0. (Dassault Systemes, Paris, France) was used for the activity coefficient calculation of polyphenols and glucosinolates in the NADESs. The software BIOVIA TURBOMOLE version 2021 (Dassault Systemes, Paris, France) was used for the geometric and energetic optimization of HBAs, HBDs, glucosinolates, and polyphenols used in this study.

### 3.2. Computational Methods

Before calculations in COSMOtherm, the geometry and charge density of all individual molecules used in this research needed to be optimized. In this work, each molecule was optimized using the COSMO-BP-TZVP template of the TURBOMOLE software package, which includes a def-TZVP basis set, DFT with the B-P83 functional level of theory, and the COSMO-RS solvation model (infinite permittivity). All calculations were performed using the software BIOVIA COSMOtherm 2020 version 20.0.0.; choline chloride salts applied as HBAs were treated as ion pairs. Organic acids were treated as protonated specimens. NADESs were treated as binary mixtures of HBDs and HBAs at a fixed stoichiometric rate. The output of the calculation is ln(*γ*) of the target compound in an NADES at 25 °C and infinite dilution with 10, 30, and 50% water (*w*/*w*).

### 3.3. NADES Preparation and Characterisation

NADESs were prepared by mixing two components in a specific molar ratio (Table 2). According to this ratio, the mass of the solvent components and the mass of water were calculated to achieve a water content of 30% (*w*/*w*). The solvent components and water were weighed and mixed in a plastic Falcon tube (V = 50 mL), and the mixture was homogenized by mixing in an orbital homogenizer at a temperature of 60 °C until a liquid, viscous, homogeneous, and transparent mixture was obtained. The prepared solvents were stored sealed at room temperature until use. The properties of the prepared NADESs (pH, density and viscosity) were determined at 25 °C. The pH values of the prepared NADESs were measured using a pH glass electrode (Mettler Toledo, Greifensee, Switzerland). The density was determined using the pycnometric method and the viscosity using a rotary viscometer (Anton Paar ViscoQC 300, Ashland, VA, USA). The polarity of DESs was determined using Nile red as an indicator following the method of [56]. All measurements were performed in triplicate.

### 3.4. Solid–Liquid Extractions

In a plastic Falcon tube, 1 g of broccoli powder and 10 mL of pre-prepared NADES were added. The mixture was heated at 75 °C for 5 min for enzymatic inactivation, followed by ultrasound treatment (100 W), then maintained at a constant temperature of 65 °C for 50 min. Subsequently, vacuum filtration was conducted using a Büchner funnel, and the filtrate was collected and stored for further analysis. The same procedure was performed using a reference solvent of 70% ethanol (*v*/*v*).

### 3.5. Determination of Total Phenolic Content

The total phenolic content (TP) was determined using the Folin–Ciocalteu method, following the protocol outlined by [57]. Briefly, the glucosinolate-rich extracts were diluted 20 times with distilled water. Then, 250 µL of the extract and 1.25 mL of 10 × diluted Folin–Ciocalteau reagent were pipetted into glass test tubes. The samples were then vortexed and incubated for 5 min at room temperature. After incubation, 1 mL of sodium carbonate solution (75 g L^−1^) was added. The samples were vortexed and then incubated in a water bath at 50 °C for 5 min. The reaction was abruptly stopped by placing the samples in an ice bath. Absorbance was measured at 760 nm, and the results were expressed as milligrams of gallic acid equivalent per gram of grape pomace (mg GAE g^−1^). All measurements were performed in triplicate. To account for any background interference from NADES components in the extracts, the corresponding NADESs were used as blanks.

### 3.6. Determination of Individual Aliphatic and Indole Glucosinolates

The determination of glucosinolates was carried out according to the international standard ISO method [58].

#### 3.6.1. Desulfation of Prepared Glucosinolate-Rich Extracts Using the Enzyme Sulfatase

The desulfation of the prepared extracts was carried out using pre-prepared columns for desulfation. First, 0.7 mL of the ion exchanger Fast DEAE Sepharose CL-6B (Cytiva, Marlborough, MA, USA) and 2 mL of imidazole formate solution were added to the column. After all the imidazole formate had drained, 1 mL of distilled water was added twice to the column. Once the water had drained, 1 mL of the appropriate extract and 1 mL of acetate buffer (0.02 M) were added twice to each column. After all the buffer had drained, 100 µL of sulfatase, an enzyme that converts glucosinolates into their desulfated form, was added. The sulfatase was left to act for 18 h at room temperature. Elution was performed with 1.5 mL of distilled water, and the eluates were collected in new, clean test tubes. The obtained eluate was analyzed by high-performance liquid chromatography (HPLC).

#### 3.6.2. High-Performance Liquid Chromatography of Desulfo-Glucosinolates

Desulfo-glucosinolates were identified and quantified using an HPLC system (1260 Infinity II, Agilent, Santa Clara, CA, USA) equipped with a diode array detector (UV/DAD, 1260 Infinity II, Agilent, USA) and an automatic sampler (1260 Infinity II, Agilent, USA). Separation was achieved on a Poroshell 120 SB C18 column (150 mm, 4.6 mm, 5 µm; Agilent, USA) using deionized water (A) and acetonitrile (B, γ = 0.20) as the mobile phases. Gradient elution was modified as follows: 0–1 min 100% A, 1–20 min a linear gradient from 100% A to 100% B, 20–25 min a linear gradient back to 100% A, followed by column equilibration from 25 to 30 min at 100% A. The flow rate was set to 1 mL min^−1^. The sample injection volume was 15 µL, and all samples were filtered through 0.45 µm polytetrafluoroethylene (PTFE) filters before injection. The column temperature was maintained at 30 °C. UV-DAD acquisitions were performed at 229 nm for desulfo-glucosinolate detection.

The retention times and spectral data of the desulfo-glucosinolates were compared with external standards. Sinigrin was used as the external standard for quantification. Quantification was performed using calibration curves of sinigrin over a concentration range of 10–1000 mg L^−1^ at 229 nm. HPLC analyses were conducted in triplicate, and the desulfo-glucosinolate content was expressed as mg of compound per gram of dry weight (dw).

### 3.7. Oxygen Radical Absorbance Capacity Assay (ORAC)

An oxygen radical absorbance capacity (ORAC) assay was conducted following the methodology outlined by [11]. The results were expressed as relative ORAC values (Equation (1)). In brief, measurements were performed in a 3 mL reaction mixture consisting of 2.25 mL of fluorescein sodium salt (0.04 μmol L^−1^) in a sodium phosphate buffer (0.075 M, pH 7.0) and 0.375 mL of diluted extracts, with Trolox (25 μmol L^−1^) serving as the standard and 0.075 M sodium phosphate buffer (pH 7) as a blank control. Following a 30 min incubation at 37 °C, 0.375 mL of AAPH was added to initiate the reaction. Fluorescence was measured every minute until the signal reached zero using a Varian Cary Eclipse Fluorescence Spectrophotometer (Palo Alto, CA, USA) with excitation at 485 nm and emission at 520 nm. The results were analyzed by calculating the differences in the areas under the fluorescein decay curve between the blank and the sample. Data were reported as mean values (*n* = 3) and expressed in µmol Trolox equivalents per gram of extract (μmol TE g^−1^). The relative ORAC value (μmol TE g^−1^) was calculated according to Equation (1):

(1) $$Relative\;ORAC\;value=(\frac{AUCs-AUCb}{AUCtrx-AUCb})\times k\times \alpha \times h$$

where *AUCs* is the area under the curve of sample, *AUCb* is the area under the curve of blank, *AUCtrx* is the area under the curve of Trolox, *k* is the dilution factor, *α* is the molar concentration of Trolox, and *h* is the ratio of the volume to the mass of the sample.

### 3.8. Determination of Biological Activity

The biocompatibility of broccoli extracts prepared in NADESs was assessed in vitro against adherent normal human keratinocytes (HaCaT) using the CellTiter 96^®^ AQueous One Solution Cell Proliferation (MTS) assay. HaCaT cells were cultured in DMEM supplemented with 10% FBS and maintained in a humidified atmosphere (5% CO₂) at 37 °C. For cytotoxicity testing, cells were seeded in 96-well plates at a density of approximately 3 × 10^4^ cells per well in 100 µL of medium and incubated for 24 h before treatment. Broccoli extracts, diluted in culture medium, were applied to cells at final concentrations of 0.5%, 1.5%, 2.5%, and 5% (*v*/*v*). Untreated cells served as controls. Following 72 h of treatment, 10 µL of MTS reagent was added per well, and cells were incubated for an additional 4 h. Absorbance at 490 nm was measured using a SpectraMax ABS Plus microplate reader to determine cell viability, expressed as a percentage of treated versus control cells. Each treatment concentration was tested in triplicate, and results are reported as mean ± standard deviation (S.D.).

### 3.9. Scratch Test

The scratch test, commonly employed to assess the effects of various factors on cell migration and wound healing, was used to evaluate the impact of B:Glc_1:1_-based broccoli extracts on HaCaT cells [53]. In this assay, HaCaT cells were seeded in 24-well plates at a density of 5 × 10^5^ cells per well in 0.5 mL of medium and incubated for approximately 24 h to form a monolayer. A scratch was then made in the center of each well using a pipette tip to create a controlled gap, simulating a wound. Initial images of the “wound” were taken with a microscope camera, after which the plates were treated with either the B:Glc_1:1_ extract, B:Glc_1:1_ alone, or left untreated as controls. The plates were incubated for 24 h, during which cell migration and growth at the scratch site were monitored by capturing images with a microscopic camera, and the ImageJ software (v1.54g, [59]) was used to measure the distance between the edges of the created gap to assess wound closure. The degree of healing and cell migration was quantified as the ratio of the average gap width before and after treatment.

## 4. Conclusions

This study demonstrates the effective use of NADESs to obtain high-value, stable liquid broccoli extracts rich in polyphenols and glucosinolates, while ensuring economic and environmental sustainability. Predictive modeling with the COSMOtherm software guided the selection of five betaine-based NADESs, resulting in extracts with significantly higher concentrations of bioactive compounds and enhanced antioxidant activity compared to ethanol extracts. Stability assessments confirmed the longevity of bioactive compounds within certain extracts. In vitro tests indicated that the NADES extracts, particularly those with glucose, mildly stimulated HaCaT cells and promoted the wound-healing process, suggesting potential benefits for skin-related applications. Furthermore, the extraction process was briefly validated as both green and cost-effective. Overall, this study advocates for the use of ready-to-use NADES-based plant extracts as a promising approach for the sustainable extraction of bioactives for potential applications in the health care industry.

## Figures and Tables

**Table 1:** Predicted ln(*γ*) values for quercetin, ferulic acid, and glucoraphanin in the selected NADESs using COSMOtherm.

		Water Content (%, *w*/*w*)											Water Content (%, *w*/*w*)										
		10	30	50	10	30	50	10	30	50			10	30	50	10	30	50	10	30	50		
NADES	Molar Ratio	Quercetin			Ferulic Acid			Glucoraphanin			NADES	Molar Ratio	Quercetin			Ferulic Acid			Glucoraphanin				
**B:CA**	**1:1**										**ChCl:Sol**	**2:3**											
**B:Glc**	**5:2**										**ChCl:Sor**	**1:1**											
**B:Glc**	**1:1**										**ChCl:U**	**1:2**											
**B:Gly**	**1:2**										**ChCl:U:Gly**	**1:2:2**											
**B:OxA:Gly**	**1:2:1**										**ChCl:Xyl**	**2:1**											
**B:Ma**	**1:1**										**ChCl:Xyol**	**5:2**											
**B:Ma:Glc**	**1:1:1**										**CA:Fru**	**1:1**											
**B:Ma:Pro**	**1:1:1**										**CA:Glc**	**1:1**											
**B:Arg**	**1:1**										**CA:Glc:Gly**	**1:1:1**											
**B:His**	**1:1**										**CA:Sor**	**2:3**											
**B:Lys**	**1:1**										**CA:Suc**	**1:1**											
**B:Xyl**	**1:1**										**Fru:Glc:U**	**1:1:2**											
**B:Suc**	**4:1**										**Glc:EG**	**1:2**											
**ChCl:CA**	**2:1**										**Glc:Fru**	**1:1**											
**ChCl:CA**	**1:1**										**Gly:Glc**	**2:1**											
**ChCl:Fru**	**1:1**										**Ma:Fru**	**2:1**											
**ChCl:Glc**	**2:1**										**Ma:Fru:Gly**	**1:1**											
**ChCl:Glc**	**1:1**										**Ma:Glc**	**1:1:1**											
**ChCl:Gly**	**1:2**										**Ma:Glc:Gly**	**1:1**										**Legend**	**ln(*γ*)**
**ChCh:Ma**	**1:1**										**Ma:Sor:Gly**	**1:1:1**											**>2**
**ChCh:Mal**	**4:1**										**Ma:Suc**	**1:1:2**											**0–2**
**ChCl:OxA**	**1:1**										**Pro:Fru:Gly**	**2:1**											**−3–0**
**ChCl:Pro:Ma**	**1:1:1**										**Pro:Ma**	**1:1:1**											**−6–(−3)**
**ChCl:Suc**	**2:1**										**Suc:Glc:Fru**	**1:2**											**−10–(−6)**
**ChCl:Sol**	**1:1**										**Suc:Glc:U**	**1:1:1**											**<−10**

Arg: arginine; B: betaine; CA: citric acid; ChCl: choline chloride; Fru: fructose; Glc: glucose; Gly: glycerol; His: histidine; Lys: lysine; MA: malic acid; Mal: malonic acid; OxA: oxalic acid; Pro: proline; Suc: sucrose; Sol: sorbitol; Sor: sorbose; U: urea; Xyl: xylose; Xyol: xylitol.

**Table 2:** Physicochemical properties and cytotoxicity of the prepared NADESs.

NADES	Molar Ratio	Viscosity [mPa·s]	Density [g cm^−3^]	pH	Polarity [kcal mol^−1^]	HaCaT EC_50_ [mg L^−1^]
B:Glc_1:1_	1:1	71.49	1.20	7.24	49.21	>2000
B:Glc_5:2_	5:2	53.45	1.20	7.85	49.90	>2000
B:Suc	4:1	54.13	1.19	7.71	49.90	>2000
B:Gly	1:2	25.70	1.17	7.03	49.72	>2000
B:MA	1:1	61.18	1.24	3.24	49.21	>2000

**Table 3:** Green credentials and solvent cost estimation (0.1 L scale) of conventional (EtOH-assisted) and NADES (B:Glc_1:1_)-assisted extraction of polyphenols and glucosinolates from broccoli.

Extraction Procedure	Yield		E-Factor ^A^ (kg kg^−1^)	EQ ^B^ (kg kg^−1^)	PMI ^C^ (kg kg^−1^)	Solvent Cost ^D^ (EUR kg^−1^)
	Polyphenols (mg g^−1^_dw_)	Glucosinolates (mmol kg^−1^_dw_)				
Conventional extraction	1.9	9.3	800	800	2000	160
NADES-assisted extraction (ready-to-use extract)	4.9	9.6	0	0	1100	140

^A^ The ratio of the weight of generated waste to the total weight of the end product (broccoli residues not included). ^B^ E-factor multiplied by an environmentally hazardous quotient. ^C^ The ratio of the total mass used in a process and the mass of the end product. ^D^ Solvent cost according to https://www.sigmaaldrich.com (accessed on 15 July 2024).

**Table 4:** SWOT (strengths, weaknesses, opportunities, threats) analysis of NADES-assisted preparation of ready-to-use plant extracts.

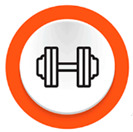	**STRENGTHS**
	**Flexibility:** possibility of the adjustment of NADES properties according to specific requirements, such as solubility and stability of various target components, toxicity, and biodegradability. **Biocompatibility:** NADES often originate from natural sources and consist of biocompatible components, making them safe for human use and the environment. **“Active” Solvent:** Since NADES components often possess biological activity (e.g., antioxidant and antimicrobial properties), they can provide added value to the products they are included in. **Unique Phytochemical Profiles:** The use of NADES enables the preparation of extracts with unique phytochemical profiles.
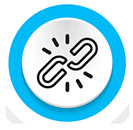	**WEAKNESSES**
	**Complexity of Formulation:** Balancing components and water in NADES formulation requires precise control and understanding of chemical interactions, which can be challenging. **Limited Stability:** Some NADES formulations may exhibit limited stability under certain storage conditions, potentially resulting in phase separation or degradation of active compounds over time, such as the negative impact of low pH in acidic NADES. **Challenges in Scale-Up:** Transitioning the preparation and use of NADES from laboratory to industrial scale, due to high viscosities and densities of NADES, can pose challenges in maintaining the consistency and quality of the final product.
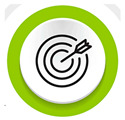	**OPPORTUNITIES**
	**Expanding Applications:** Collaboration between the academic community and institutions across various research fields could lead to broader applications of NADES in production and industrial development. **Regulatory Approvals:** Obtaining regulatory approvals could enhance the commercialization of these natural solvents, creating opportunities to open new markets. **Enhanced Characterization:** Increased application of NADES could potentially allow for more detailed characterization of known plant metabolites and the discovery of new plant properties.
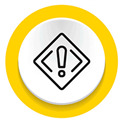	**THREATS**
	**Increasing Competition:** A more competitive research landscape due to rivalry among investors. **Regulatory Barriers:** Strict safety, quality, and efficacy requirements for NADES before potential market entry.

## Data Availability

The original contributions presented in this study are included in the article. Further inquiries can be directed to the corresponding author.
